# Audiovisual Delay as a Novel Cue to Visual Distance

**DOI:** 10.1371/journal.pone.0141125

**Published:** 2015-10-28

**Authors:** Philip Jaekl, Jakob Seidlitz, Laurence R. Harris, Duje Tadin

**Affiliations:** 1 Center for Visual Science and Department of Brain and Cognitive Sciences, University of Rochester, Rochester, New York, United States of America; 2 Laboratory of Brain and Cognition, National Institute of Mental Health, National Institutes of Health, Bethesda, Maryland, United States of America; 3 Centre for Vision Research and Departments of Psychology, Biology and Kinesiology, York University, Toronto, Ontario, Canada; 4 Department of Ophthalmology, University of Rochester School of Medicine, Rochester, New York, United States of America; Ecole Normale Supérieure & CNRS, FRANCE

## Abstract

For audiovisual sensory events, sound arrives with a delay relative to light that increases with event distance. It is unknown, however, whether humans can use these ubiquitous sound delays as an information source for distance computation. Here, we tested the hypothesis that audiovisual delays can both bias and improve human perceptual distance discrimination, such that visual stimuli paired with auditory delays are perceived as more distant and are thereby an ordinal distance cue. In two experiments, participants judged the relative distance of two repetitively displayed three-dimensional dot clusters, both presented with sounds of varying delays. In the first experiment, dot clusters presented with a sound delay were judged to be more distant than dot clusters paired with equivalent sound leads. In the second experiment, we confirmed that the presence of a sound delay was sufficient to cause stimuli to appear as more distant. Additionally, we found that ecologically congruent pairing of more distant events with a sound delay resulted in an increase in the precision of distance judgments. A control experiment determined that the sound delay duration influencing these distance judgments was not detectable, thereby eliminating decision-level influence. In sum, we present evidence that audiovisual delays can be an ordinal cue to visual distance.

## Introduction

Perceiving the distance to objects is a key function of sensory systems. In humans, distance perception is thought to rely primarily on visual cues [1] while free field auditory distance estimation is regarded as considerably less reliable [2]. Moreover, distance estimation is most often conceptualized as a self-contained, unisensory process, ignoring the fact that many events provide both auditory and visual signals. In such cases, light arrives essentially instantaneously whereas sound arrives after a delay that varies with the distance between the observer and the source event. This crossmodal asynchrony has been considered an impediment to correctly perceiving ‘bound’ auditory and visual components caused by the same source event, requiring compensation for veridical perception. Some investigations have demonstrated that such compensation can occur [3–5]. Compensation does not occur when temporal delays are too small and/or visual distance information is not easily available [6–8]. However, regardless of whether the delay compensation takes place, asynchronies in audiovisual arrival times contain temporal information that can reliably signal the distance of the events.

Previous findings demonstrating neural selectivity to audiovisual sound delays [9,10] are consistent with behavioral demonstrations that perception of audiovisual simultaneity at source can be robust to sound delays [3] and can even take into account the dependency of sound delays on event distance as derived by visual distance cues [4,5,11]. Here, we ask whether our sensory systems go beyond simply correcting for audiovisual delays and use such asynchronies as an information source for distance computation. This question was motivated by our recent results showing that audiovisual delays can affect perceived stimulus size, indirectly suggesting a role of audiovisual asynchrony in size/distance scaling [12]. In brief, we found that when a circle was shown in an otherwise dark room, it was perceived as being larger when presented with short sound delays. No effects of sound on perceived size were found for synchronous or leading sounds. One possible explanation of this finding is that sound delays cause objects to appear farther and thereby larger. Here, we aim to test this hypothesis and determine if sound delays can indeed influence perceived distance.

Broadly, there are two different kinds of visual distance cues, based on the scale of information they provide: metric and ordinal. Metric cues, such as stereopsis, signal precise, interval-ratio type information about object distance. In contrast, ordinal cues, such as occlusion and aerial perspective, only provide ordinal or simply modulatory information regarding visual distance [13,14].

There is evidence that some animals, notably bats and dolphins, can use sound delays as a metric distance cue. There are even documented cases of blind humans who exhibit coarse forms of echolocation [15–17]. However, it is unlikely that humans, with our less advanced auditory systems, can typically exhibit same levels of echolocating sophistication as animals whose predominant sensory system is echolocation [18]. Moreover, in free field environments sound delay varies with wind speed and direction, temperature and humidity [19]–factors further complicating the use sound delays as a precise, metric cue to distance in such environments (less open environments such as forests or caves may provide additional auditory distance cues via direct-to reverberant energy ratios [20]). This, however, does not preclude human utilization of audiovisual delays as ordinal information about event distance—a hypothesis tested in this study. As an ordinal distance cue, sound delays would not provide precise distance information but rather work in combination with other depth cues to help improve distance perception. Specifically, we tested the hypothesis that the presence of sound delays will affect the perceived visual distance of audiovisual events by effectively pulling the perceived stimulus location toward more distant locations indicated by sound delays. Moreover, if sound delays do play a role in human distance perception, the presence of sound delays that are congruent with other distance cues should increase the precision of distance estimation [21,22]. Note that here we are concerned with perceptual effects of relatively brief sound delays, on the order of tens of milliseconds. Considerably longer sound delays, such as those associated with lightning strikes, can also signal event distance, but such effects are cognitive in nature.

## Experiment 1

**Fig 1:**
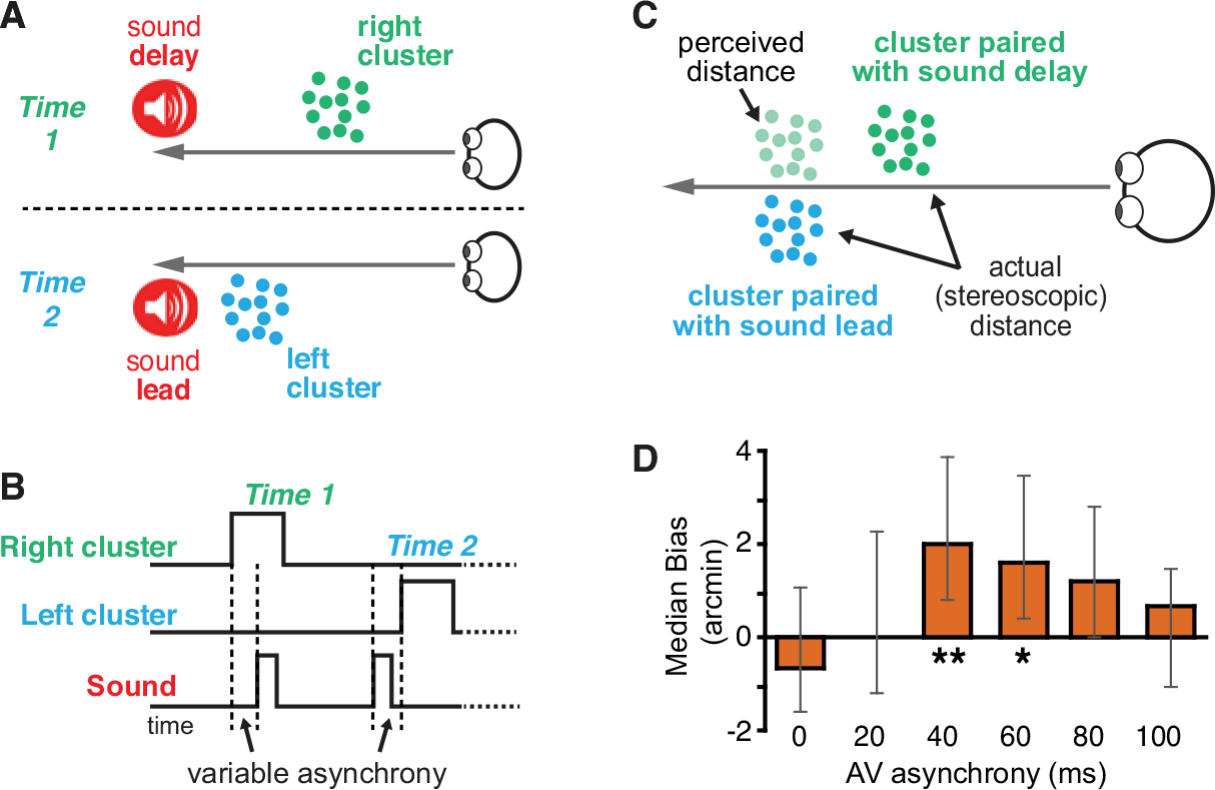
Experiment 1. (A) Stimulus timeline. Right and left clusters alternated continuously, appearing for 225 ms with a 600 ms inter-stimulus interval. Sounds paired with one cluster preceded visual onset while sounds paired with the other cluster were delayed by an equal amount that ranged between 0 and 100 ms. Time one and time two are illustrated in a spatial manner in 1B. (B) Spatial arrangement of alternating dot clusters as conveyed by stereoscopic depth. At ‘Time 1,’ the right cluster was presented at a stereoscopically defined distance and paired with a sound delay. At ‘Time 2’ after an interstimulus interval, the left cluster was presented at a different distance (here shown as more distant) and paired with a sound lead. Participants adjusted the relative distance of two alternating dot clusters until they appeared to be at the same perceived distance. (C) Experimental rationale. If the presence of sound delays increases perceived visual distance, the cluster presented with a sound delay would need to be shown physically closer for the two clusters to appear equidistant. (D) Averaged median adjustments of stereo disparity. Positive biases indicate that dot clusters presented with sound delays were perceived as more distant than clusters with sound leads (**p = 0.015, *p = 0.048). Error bars are 95% confidence intervals from the bootstrap analysis as described in Materials and Methods.

To explore whether audiovisual asynchronies can bias visual distance judgments, we asked participants to adjust the relative distance of two stereoscopically presented, three-dimensional random dot clusters (Fig 1A and 1B) to make them appear equidistant. The dot clusters were separated laterally and presented in a repetitive sequence (Fig 1A), alternating until participants were confident in each adjustment. Our method was partially inspired by Freeman and Driver [23], who used alternating audiovisual stimuli to influence spatio-temporal processing of visual motion direction. On every trial, each cluster was paired with a task-irrelevant sound. The onset of one cluster was paired with a sound delayed by between 0 and 100ms and the other was paired with an identical sound leading by the same asynchrony. We hypothesized that if the sound delay increases perceptual distance, the sound-delayed clusters would match the perceived distance of the sound-leading clusters at physically closer locations as indicated by stereo disparity (Fig 1A).

### Materials and Methods

#### Participants

Five participants (ages 21 to 35, two female and three male) completed the first experiment. All had normal or corrected-to-normal vision and were pre-screened to ensure they had acute stereopsis (< 32 arcsec, as measured using a stereo acuity test (VAC Random Dot 2, Vision Assessment Corporation) and were able to fuse stereoscopic images in the display. The participants did not have any known hearing problems. Both Experiment 1 and 2 were conducted under a protocol approved by the University of Rochester Research Subjects Review Board and included written consent from participants.

#### Apparatus

Visual stimuli were projected by a customized 120 Hz DLP projector (DepthQ WXGA-360) onto a projection screen in a dark room. The projector’s color wheel was removed, allowing the presentation of three gray-scale images per cycle at 120 Hz. Frame timing and duration were carefully verified with an oscilloscope. DLP projectors are inherently linear, and this was verified with a Minolta LS-110 photometer. The experiments were controlled by a Mac Pro 4.1 running Matlab 7.5 with the Psychophysics Toolbox [24,25].

Participants were seated at a distance of 2.4 m from the display with their heads stabilized on a chinrest. The display size was 24° wide by 14° high. Left and right-eye images were alternated by RealD CE4 (RealD, Beverly Hills, CA) liquid crystal shutter glasses at a rate of 120 Hz. Stimuli were only shown in the middle third of each 8.3 ms frame, leaving 5.6 ms of dark between each stimulus frame and enabling good stereo separation. Sounds were presented using two PC speakers symmetrically flanking the visual display, separated by 1 m. Thus, the horizontal and vertical coordinates of the perceived stereo sound location were designed to approximate the visual stimulus position—a stimulus property this is known to facilitate multisensory interactions [26].

#### Stimuli and Procedure

Dots were shown in two stereoscopically arranged clusters. Both consisted of 50 non-overlapping white dots (dot size .07°, luminance 115 cd/m^2^) spread randomly in two cylindrical ‘volumes’ (diameter = 1.5°), centered 0.5° to the left and right of display center. When both clusters were shown at the same simulated distance, each cluster was shown at a 1.3° cyclopean reference disparity corresponding to a distance of about 2.7 m from the participant, varying slightly with interocular distance. The clusters spanned a range of about 3.25 cm depth (given by disparity). All stimuli were presented on a grey background (30 cd/m^2^). This large diffuse background made it difficult to use relative distance information from objects in the room as an aid to correct responses. Sounds consisted of 5 ms ‘click’ noises, presented at approximately 73 dB (http://www.freesound.org/people/junggle/sounds/28812/). These 'click' sounds had multiple frequency peaks that declined in power nearly monotonically between 0 and 200 Hz.

The left and right clusters were presented alternately in a repetitive sequence, each cluster appearing for 225 ms with 600 ms between onsets (Fig 1A). These timing parameters were determined during experiment piloting and set to mitigate the perception of apparent motion between the dot clusters. Each trial started with one cluster (left or right, chosen randomly) positioned closer than the 1.3° reference disparity and the other further by the same, randomly chosen distance. Participants were instructed to match the perceived distance of the clusters as they continuously alternated on the display (Fig 1B). Pressing the left arrow increased the disparity (and perceived distance) of the left cluster while decreasing the disparity of the right cluster by an equal increment (0.02°). A small flash indicator informed the observer that the ‘distance’ had changed. Pressing the right arrow resulted in the opposite disparity change. When the participants were confident that the dot clusters appeared to be the same distance they pressed the down arrow and the difference between the mean cyclopean disparity of the left and right clusters was recorded as their adjustment value. Participants were advised to carefully make the adjustments, viewing the alternating left and right stimuli until they could confidently gauge their relative distance.

Task-irrelevant ‘click’ sounds were presented with an asynchrony relative to the visual onsets of each cluster, ranging between 0 ms and 100 ms in increments of 20 ms. For half the trials (randomly selected), sound onset was delayed relative to the visual onset of the left cluster and led the onset of the subsequently appearing right cluster. The remaining trials had the opposite left/right arrangement.

Three of the participants completed three experimental sessions. In each session the adjustment task was repeated 3 times when the delay was 0 ms and three times for each asynchrony (20, 40, 60, 80, 100 ms). Two subjects completed an additional session, yielding 3 more adjustments when the delay was 0 ms and at each asynchrony.

#### Analysis

For each participant, we computed the median adjustment value for each sound delay side (left and right) in each asynchrony condition, and used the difference of these left/right results for each sound delay, yielding bias results shown in Fig 1C. To compute statistical significance for each asynchrony, we drew 10,000 bootstrap samples from each participant and re-computed bias results as described above. Significance was calculated based on the proportion of the samples where the computed bias was less or equal to 0.

We used median adjustment values as some participants occasionally had very large adjustment results, thereby accruing a skewed distribution. Repeating the same bootstrap analysis with mean adjustment values (instead of using medians) also yielded significant biases at 40 and 60 ms asynchrony (*p* = 0.0004 and 0.0001, respectively), matching the median analysis reported below. Data for experiment one are available at the following URL: http://figshare.com/articles/_Jaekl_et_al_2015_Audiovisual_delay_as_a_novel_cue_to_distance_Exp_1_data/1494822

### Results and Discussion

The task-irrelevant sound asynchrony had significant modulatory effects on median distance adjustments (Fig 1C): for sound asynchronies of 40 and 60 ms. Dot clusters paired with delayed sounds perceptually matched the sound-leading clusters at physical locations cued by disparity that were physically closer (40 ms delay:*p* = 0.015, 60 ms delay: *p* = 0.048). These results suggest an influential role for audiovisual asynchronies in the computation of visual distance. The present experiment, however, cannot unequivocally determine whether this result was carried by sound delays or sound leads as both asynchrony types were shown together. Because of the ecological validity of audiovisual delays (for an audiovisual event with concurrent stimulus onsets, sound arrival at the ear is always delayed relative to the visual stimulation of the retina) and our previous findings showing no effect of sound leads on size/distance scaling [12], we hypothesized that perceived distance was specifically affected by sound delays. This hypothesis was tested directly in Experiment 2.

## Experiment 2

**Fig 2:**
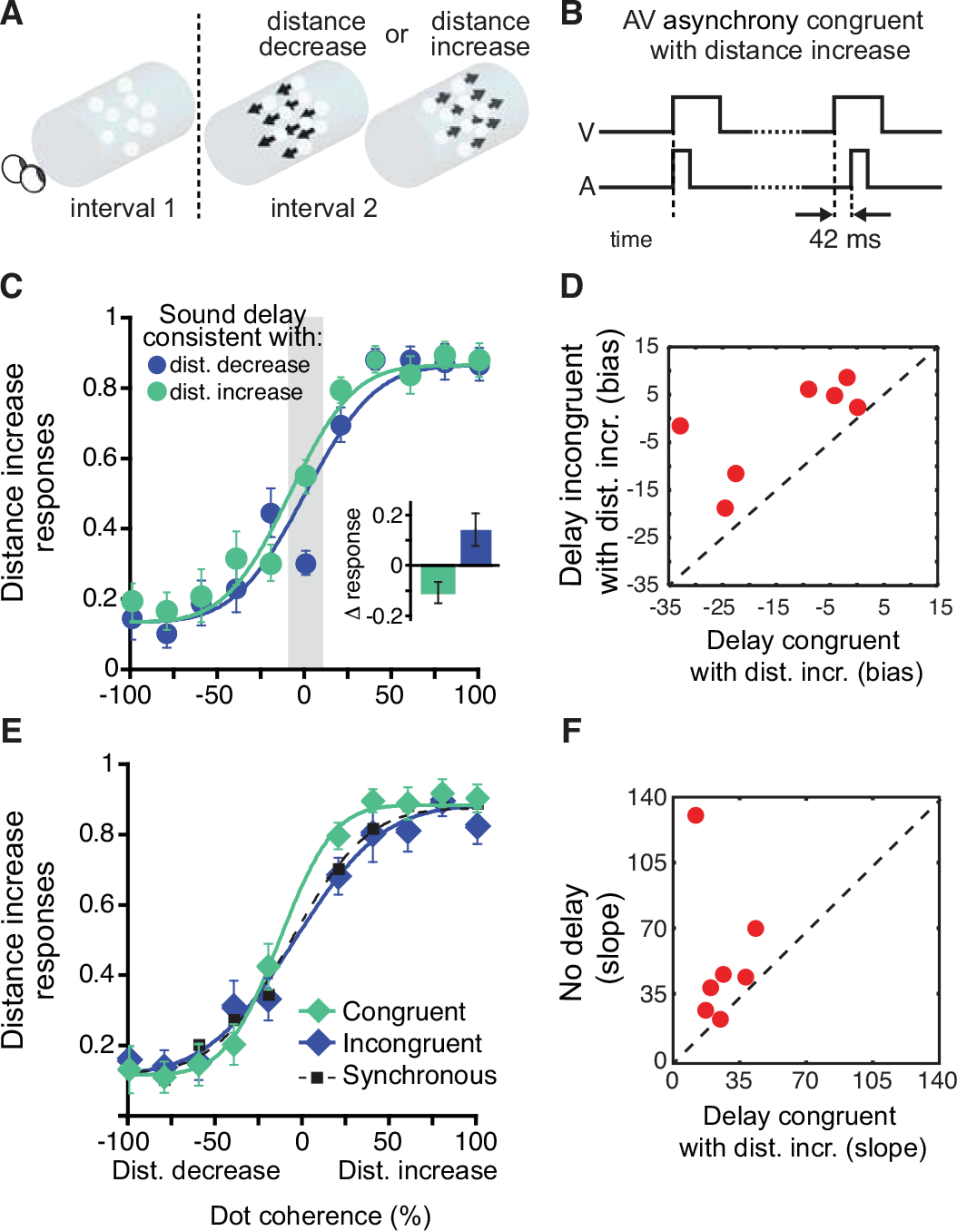
Experiment 2. (A) Experimental design. In the first interval, a dot cluster was presented at the reference distance. In the second interval, the disparity of the dot cluster was either increased or decreased, corresponding to a distance change of ~13 cm. The illustration shows shifts at 100% coherence. (B) Stimulus timeline. Sounds were either synchronous with the onset of the visual stimulus (first period in this example) or delayed by 42 ms (second period). Shown here is a trial in which the sound delay is consistent with a visual distance increase. (C) Bias. Proportion of times that subjects reported the second stimulus as being more distant, plotted as a function of dot coherence. Trials in which sound delays were consistent with distance increase are shown in light green symbols while trials consistent with a distance decrease are shown in dark blue symbols. Curves show psychometric functions fitted to the data. Participants were more likely to perceive a distance increase when sound delays were presented in the second interval (i.e., ecologically consistent with a distance increase). Inset shows changes in percent response to ambiguous (i.e., incoherent) stimuli (shaded area) plotted relative to the results of the audiovisually synchronous control condition. Error bars are SEM. (D) Data for individual participants (shown as averaged in panel C). Bias terms of psychometric functions (i.e., Gaussian mean). This plot contrasts performance in trials when sound delays were consistent with distance increases with those consistent with distance decreases (units are % coherence). Points above the unity line indicate bias towards perceiving a distance increase when the delay occurred in the 2^nd^ interval. (E) Precision. Same data as in panel C but divided into the conditions where visual and auditory cues were congruent (light green diamonds) or incongruent (dark blue diamonds). The synchronous control condition is plotted as small black squares. (F) Data for individual participants (shown as averaged in panel E). Slope terms of psychometric functions (i.e., Gaussian standard deviation). This plot contrasts data from trials with ecologically congruent stimulus-delay pairings with data from the synchronous condition (units are % coherence). Points above the unity line indicate higher precision when visual distance changes were congruent with distance changes implied by sound delays.

Next, we conducted an experiment to rigorously test the effect of sound delays on both the *bias* and *precision* of distance judgments. Specifically, we aimed to replicate the asynchrony bias found in Experiment 1 and, more importantly, determine if ecologically valid sound delays can actually improve the precision of visual distance judgments. For this experiment, we focused on audiovisual delays of 42 ms—a value between two delays with significant results in Experiment 1, but closer to the 40 ms delay that yielded the largest effect. Each trial consisted of two intervals. In the first, the mean stereo disparity of a single dot cluster was held constant (Fig 2A). In the second interval, by changing the disparity, the dot cluster was displaced either away from or toward the observer (Fig 2A). Participants discriminated this distance change for a range of dot coherences (0–100%)—an approach analogous to the widely used ‘motion coherence’ task [27]. At 100% coherence, the entire cluster was displaced rigidly (Fig 2A). At lower coherences, the coherent dots were shifted rigidly while the remaining dots were assigned a random disparity within the same volume. As in the first experiment, these stimuli were paired with task-irrelevant sounds. In one randomly chosen interval audiovisual onsets were synchronous, and in the other sound onset was delayed by 42 ms (Fig 2B), corresponding to an audiovisual delay associated with a more distant physical event.

### Method

#### Participants

Seven (ages 21 to 37, four male and three female) new participants completed the second experiment. As in the first experiment, participants were pre-screened to ensure they had acute stereopsis (< 32 arcsec, as measured using a stereo acuity test (VAC Random Dot 2, Vision Assessment Corporation) and were able to fuse stereoscopic images in the display.

#### Apparatus

Participants were seated at a distance of 1.5 m with the display size 37° wide by 21° high. Other apparatus details were as described for Experiment 1. Importantly, the horizontal and vertical coordinates of the perceived sound location were designed to approximate the visual stimulus position.

#### Stimuli and Procedure

Two stereoscopically displayed dot clusters were presented in sequence and consisted of 218 non-overlapping white dots within a single cylindrical ‘volume’ (visual diameter = 2.5°) centered on the screen. As in experiment one, the luminance of the dots was 115 cd/m^2^ and the background was 35 cd/m^2^. The left and right eye dots for each cluster ranged over 0.08° cyclopean disparity, corresponding to a cluster length in depth of about 6 cm. A fixation crosshair at the center of the display (diameter 0.5°) was used to initiate each trial of the experiment. The fixation crosshair was displayed stereoscopically (0.6° cyclopean disparity; corresponding to a simulated distance of about 1.8 m), always positioned at the distance corresponding to the mean disparity of the first dot cluster (i.e. at 1.8 m). The same 5 ms ‘click’ sounds used in experiment one were used in experiment two.

Before the experimental sessions, participants completed two practice sessions. For the first, they adjusted the mean disparity between two sequentially viewed dot clusters with unlimited duration, to familiarize themselves with the stimuli. Here, the displacement (100% coherent) occurred when the participant pressed the spacebar of the keyboard. The second practice session involved the same task as the main experiment but we used only high coherence levels (60, 80 and 100%) and only synchronous audio-visual onsets (i.e., key experimental manipulations were not used). If a participant could not meet a criterion of a mean of 80% correct performance for these highly coherent stimuli, they did not participate in the main experiment. Six new potential participants were excluded by this criterion and did not participate in the main experiment. Note that this exclusion procedure was not based on participants’ performance in key experimental conditions (as described below). Rather, it was implemented after our pilot work that revealed that a proportion of potential participants exhibited difficulty with perceiving stereoscopic stimuli when even a small amount of noise was added to the displays.

A two-interval forced choice task (2IFC) was used, with each dot cluster accompanied by an uninformative sound. Each trial began with the fixation crosshair displayed for 33 ms. After this duration a blank screen was displayed for a random duration of between 750 ms and 1 s, followed by the onset of the first dot cluster (duration 125 ms). The second cluster (duration 125 ms) was presented after a random inter-stimulus interval of between 830 ms and 1.25 s. As in the first experiment, this large inter-stimulus interval mitigated apparent motion cues. The first cluster was always centered in depth on the fixation distance (1.8 m), effectively constituting a standard stimulus. The second cluster was presented with a shift of about ±13cm in depth (0.15° disparity).

In one interval, sound was presented synchronously with the onset of the cluster. In the other interval, the sound was delayed by 42 ms (Fig 2B). This delay was presented an equal number of times in both the first and second interval. A baseline control condition with synchronous audiovisual onsets in both intervals was also run for comparison purposes. The results from this condition were used for computing the data shown in Fig 2C (inset) and as a baseline for the congruency analysis (Fig 2E and 2F).

Task difficulty was modulated by varying the dot coherence, defined as the percentage of dots that were displaced rigidly in depth (by ±13cm) in the second interval, maintaining their relative positions. The remaining dots were displaced randomly by up to ±26cm. Coherence was varied between 0% and 100%, in steps of 20%. Participants were instructed to judge the direction of displacement of the dot cluster—i.e., whether the second cluster appeared *further* or *closer* than the first cluster. They indicated their choice by pressing one of two keys: left arrow for further and right arrow for closer. Each coherence level was repeated 20 times for both displacement directions and each sound delay condition (first interval, second interval and synchronous). In total there were 660 trials per participant (3 sound arrangements*(2 displacement directions*5 coherence levels + 1 zero coherence level)*20 repeats).

During the debriefing session at the end of the experiment, we found that participants were unaware that sound delays occurred during the experiment—sounds were perceived as simultaneously paired with visual stimuli. This is consistent with previous findings showing a relatively wide range of asynchronies where visual and auditory stimuli are perceived as simultaneous [2,3,11]. To empirically confirm that the stimulus delays used in this experiment were not consciously discriminable, we ran a simple control experiment using stimuli identical to those used in main experiment. Participants (*n* = 7) were explicitly instructed to choose the interval containing the delay while simply “observing” the shifts in the distance of the dot clusters. Coherence levels and shift direction (closer or further) were chosen randomly for 200 trials for each participant. The results revealed near chance level performance (mean = 53.6% correct, range = 48–58.5% correct). When analyzed individually, only one participant exhibited performance that was significantly different than chance (p = 0.02, Binomial test, not corrected for multiple comparisons; all other participants p > 0.14). We note that this control experiment provides a conservative test of the discriminability of sound delays in the main experiment. There, participants’ attention was directed to the distance discrimination task they were performing and they were not informed about the presence of the sound delays. In contrast, in the control experiment, participants’ attention was explicitly directed to detecting the sound delays.

#### Analysis

The percentage of times each displacement was perceived as ‘further’ was plotted as a function of coherence level (negative coherence values represent percentage of dots shifting coherently *toward* the observer), and was fitted with a cumulative Gaussian function that also included one lapse parameter. The mean of the fitted cumulative Gaussian represents equal likelihood of perceiving the displacement in either direction, representing the point of subjective equality (PSE). To estimate the precision associated with correctly discriminating distance changes in the dot clusters (Fig 2E and 2F), the data were grouped by the (in)congruency of the visual displacements and sound delays. Specifically, a delayed sound in the second interval is congruent with a displacement of the visual stimulus away from the observer and vice versa. For this analysis, the 0% coherence points were omitted, as this arrangement provides no information about visual distance change. The slope of the fitted cumulative Gaussian was taken as the measure of the precision of visual distance judgments.

To compute statistical significance, we drew 10,000 pairs of bootstrap samples from a pooled data set that included data from all participants and fit each sample with a cumulative Gaussian function. To account for the repeated measures experimental design, each pair of bootstrap samples had identical sampling of the two conditions that were compared (Fig 2C and 2D: sound delay in interval one vs. sound delay in interval two; Fig 2E and 2F: congruent vs. incongruent trials). For the bias analysis (Fig 2C), significance was defined as the proportion of the samples where the mean of the fitted Gaussian distribution was larger for sound delays consistent with distance decrease. For the precision analyses (Fig 2E), significance was defined as the proportion of the samples where the slope of the fitted Gaussian distribution was steeper (i.e., more precise) for stimuli that had sound delays congruent with visual distance change than slopes for incongruent and synchronous stimuli. To test for the robustness of the reported results, we repeated the above-described analysis with the following changes: First, data were fitted with a cumulative Gaussian with no lapse parameters. Second, data were fitted with a cumulative Gaussian with two lapse parameters (i.e., separate floor and ceiling parameters). This did not affect the reported results (bias analysis, p = 0.02 and 0.03; slope analysis, p = 0.01 and 0.006, respectively). Data for experiment two are available at the following URL: http://figshare.com/articles/_Jaekl_et_al_2015_Audiovisual_delay_as_a_novel_cue_to_distance_Exp_2_data/1494821

### Results and Discussion

To test for a role of sound delays in biasing distance judgments, we fit the results with cumulative Gaussians and compared the distribution means between the two sound delay conditions. Individual participant means of these Gaussian fits are shown in Fig 2D. Subjects were more likely to perceive the second-interval dot cluster as displaced ‘further’ when the sound delay was presented in the second interval, consistent with the results of the first experiment (Fig 2C; p = 0.04). This effect of task irrelevant sound delays was most strongly pronounced at the ambiguous, 0% coherence level (Fig 2C inset in left panel, t_6_ = 4.89, p = 0.003).

If sound delays aid distance perception, changes in distance that provide *congruent* asynchrony cues (i.e. both modalities signal the same direction of distance change) should be judged more precisely than events for which changes in sound delay are incongruent with changes in visual distance information—in line with other studies yielding enhancements for congruent audiovisual stimuli [28–30]. To test this hypothesis, we compared congruent audiovisual trials (e.g., an increase in visual distance paired with a sound delay in the second interval) with trials containing audiovisual incongruency (e.g., a decrease in visual distance paired with a sound delay in the second interval). The cumulative Gaussian fits to the data revealed significantly higher distance estimation precision for congruent trials (Fig 2D), as evidenced by a significantly steeper slope in the congruent condition (p = 0.003). A comparison with the synchronous control condition (dotted line in Fig 2E), revealed that this effect is due to ecological congruence between delay interval and displacement direction (p = 0.004) with no corresponding differences between the incongruent and simultaneous conditions (p = 0.76). The incongruent vs. synchronous condition slope values for individual participants are shown in Fig 2F.

This pattern of results argues against the simple explanation that participants’ decisions/responses were simply inclined toward the interval with the sound delay. Specifically, as sound delays were not predictive of visual distance, such decisional tendency predicts both an enhancement for congruent audiovisual shifts and a *decrement* in performance for incongruent stimuli across the range of coherence levels. Instead, we only found that distance judgments were more precise when more distant events were accompanied with delayed sounds, a finding indicative of sensory-level changes in distance processing due to the presence of congruent sound delays. This conclusion is further supported by the results of the control experiment which confirmed that the sound delays used the main experiment (Fig 2B) were not consciously discriminable (see Method).

## General Discussion

We found that audiovisual delays can both bias visual distance judgments and improve their precision when sound delays are paired with visual stimuli in an ecologically valid manner. These perceptual changes occurred even though audiovisual delays were not overtly informative about object distance and were too brief to be consciously discriminated [9], indicating that small audio-visual time-of-arrival differences can contribute significantly to the perceived distance of events.

Our finding of strongest effects for audiovisual delays between 40 and 60 ms (Experiment 1) is consistent with neurophysiological data showing sensitivity to sound-delayed audiovisual stimuli in the superior colliculus, a midbrain region that functions imperatively for integrating auditory and visual signals for attending to and localizing audiovisual stimuli, where auditory response latencies can precede visual latencies by 40 to 70 ms (Wallace, Wilkinson & Stein, 1996; Bell, Meredith, Van Opstal, & Munoz, 2006, also see Boehnke & Munoz, 2008). Importantly, there can be considerable multisensory response enhancement within this asynchrony range (Meredith, Nemitz an Stein, 1987)—a response property that could have contributed to the present findings. Speculatively, one explanation is that for audiovisual delays to override other distance cues present in our displays (i.e., stereopsis), sound delays needed to be sufficiently long to exploit this neurophysiological sensitivity [9,10]. This account may also explain why sound delays seem to work as an ordinal rather than a metric depth cue, where a delay needs to be sufficiently long enough before it can affect perceived distance.

The sound delays found to influence distance judgments were longer than what would be expected if such asynchronies were a metric cue to distance (e.g. a 40 ms audiovisual delay would signal an event at approximately 13.6 m distance). Rather, the present data indicate that sound delays modulate perceived distance such that events paired with delayed sounds are *more likely* to be perceived as more distant, in an ordinal manner. By a way of comparison, this is similar to the well-established depth cue of aerial perspective where lower contrast objects are *more likely* to be perceived as further [1]. Our results are also in line with other studies showing that ordinal depth cues can have modulatory influence on perceived distances cued primarily by disparity [13,31–33]. Our speculation is that perceptual experience makes these ordinal cues effective in aiding distance perception; we learn that objects paired with sound delays are more likely to be distant as we learn that objects that appear obstructed are more likely to be further away. It is possible that sound delays might tend towards being a metric cue that can be used more accurately at substantially longer distances. At greater distances, other depth cues, such as stereopsis and vergence angle, become less effective while audiovisual delays become longer and thus, more discriminable. An important question for future research is whether under such conditions sound delays start to play a more dominant role in distance perception. Another important characteristic of our results is the implicit nature of depth information conveyed by audiovisual delays—subjects were unaware of the sound delays in Experiment 2. While depth information is usually explicit, there are instances where subjects exhibit unconscious processing of depth cues [34].

Here, we make an important assumption that auditory and visual stimuli in our experiments are bound and perceived as unified events. While we did not explicitly test crossmodal binding, prior research supports this assumption. Humans generally bind crossmodal stimuli that are in close spatial and temporal register [35], into unified percepts [36]. Aside from the incongruence in depth implied by the sound delays, our stimuli generally obeyed the spatial and temporal principles of sensory integration commonly thought to support crossmodal binding [37]. Spatially, the stereo position of the sound approximated the position of the visual stimuli both laterally and vertically. Temporally, the asynchronies at which perceived distances were influenced were well within the putative temporal window for audiovisual integration [38]—an assertion further supported by the control experiment data showing that participants could not consciously discriminate sound delays in Experiment 2. Taken together, this argues that auditory and visual component stimuli were perceptually bound, and thus causally inferred to the same source event. This keeps audiovisual delay duration as the principal source of information that could influence the perceived audiovisual positions.

The present findings draw comparison to the well-known study of ventriloquism by Alais and Burr [21]. The authors found that for spatially offset audiovisual stimuli, sound can both bias and improve the precision of visual position judgments. Here, we found that for spatially co-localized audiovisual stimuli, perceived as resulting from the same source event, the perceived visual distance can be thought of as ‘ventriloquised’ towards more distant locations implied by sound delays. This significant *modulatory* effect of audiovisual delays on perceived distance reveals an important role of cross-modal interactions in distance computation. Evidently, audiovisual delays can be added to the list of cues humans use to estimate event distances.
